# Acquired dural arteriovenous fistula after cerebellopontine angle meningioma: A case report

**DOI:** 10.1097/MD.0000000000029671

**Published:** 2022-07-15

**Authors:** Jung-Soo Park, Jong-Myong Lee

**Affiliations:** a Department of Neurosurgery, Jeonbuk sNational University Hospital and Medical School, Chon-Ju, Republic of Korea.

**Keywords:** arteriovenous fistula, bone defects, craniotomy, craniectomy

## Abstract

**Rationale:**

Intracranial brain surgeries, including ventriculostomy, burr hole, craniotomy, and craniectomy, are the most common causes of acquired dural arteriovenous fistula (dAVF). Here we report a case of acquired dAVF after a cerebellopontine angle meningioma surgery.

**Patient concerns:**

A 51-year-old woman was diagnosed with a 40-mm cerebellopontine angle meningioma. The patient underwent surgery via a retrosigmoid suboccipital approach. A small craniotomy and an additional craniectomy were performed. At 7 months after the surgery, she presented with pulsating tinnitus and headache.

**Diagnosis:**

Magnetic resonance imaging and digital subtraction angiography showed a dAVF that was fed by the occipital artery and drained into transverse and sigmoid sinuses.

**Interventions:**

We performed Onyx® (Irvine, CA) embolization.

**Outcomes:**

The patient’s symptoms completely improved.

**Lessons:**

Craniectomy defects, partially exposed sinuses, and incomplete cranioplasty might be risk factors for iatrogenic dAVF after a retrosigmoid suboccipital craniotomy or craniectomy. Complete reconstructive cranioplasty is an essential procedure to prevent a direct connection between the venous sinus and the external carotid artery.

## 1. Introduction

Dural arteriovenous fistula (dAVF) is a heterogeneous vascular disease. Acquired dAVFs are rare with an unknown etiology. Clinical symptoms of dAVF can vary depending on its location and hemodynamic status. Acquired dAVF is associated with cerebral venous thrombosis, sinus occlusion, trauma, and brain surgery.^[1]^ Intracranial brain surgeries including ventriculostomy, burr hole, craniotomy, and craniectomy are the most common causes of acquired dAVF.^[1]^ We report a case of acquired dAVF after a cerebellopontine angle meningioma surgery. Causes and preventive methods for dAVF after a retrosigmoid craniectomy or craniotomy are also discussed.

## 2. Case Report

**Figure 1.**
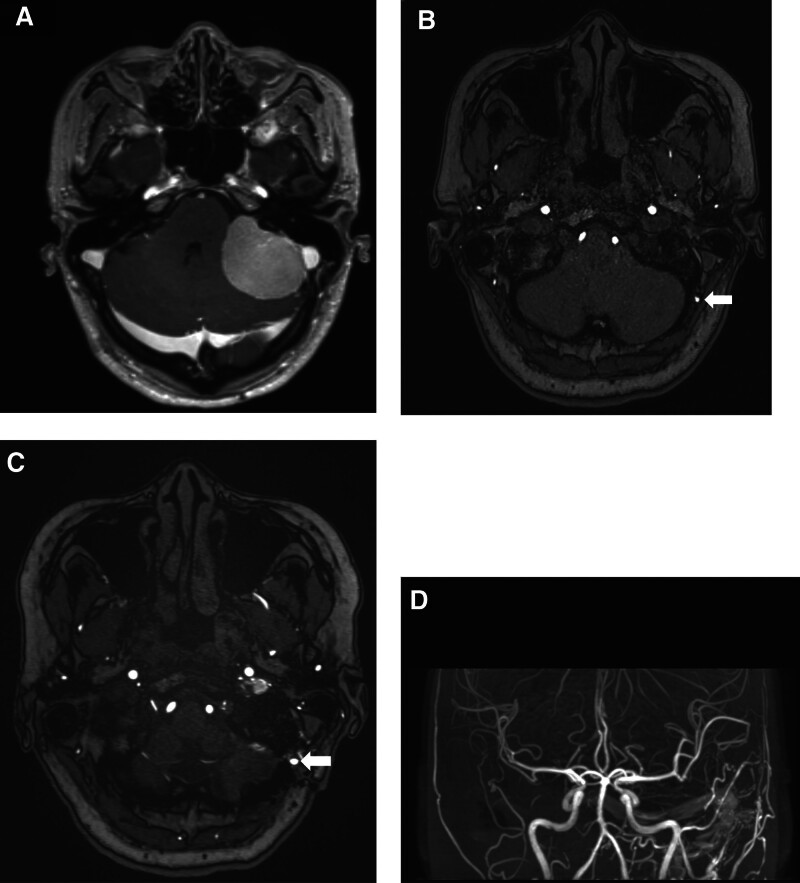
(A) Preoperative magnetic resonance imaging showing 4-cm-sized cerebellopontine angel meningioma. (B) Preoperative noncontrast-enhanced time-of-flight–magnetic resonance imaging showing a normal occipital artery (arrow). (C) Postoperative noncontrast-enhanced time-of-flight–magnetic resonance imaging showing a dilated occipital artery (arrow). (D) Postoperative magnetic resonance angiography showing arterialized transverse and sigmoid sinuses.

**Figure 2.**
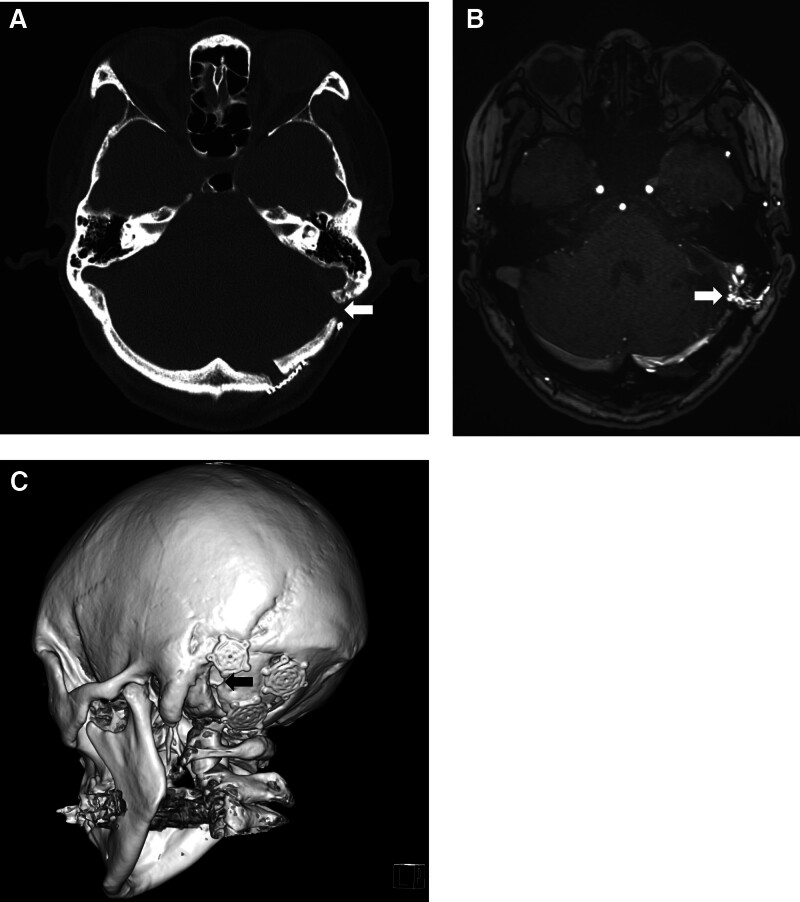
(A) Postoperative computerized tomography scan showing incomplete cranioplasty and remaining bone defect (arrow). (B) Noncontrast time-of-flight–magnetic resonance imaging showing that numerous branches of the occipital artery are directly connected to the sigmoid sinus through the bone defect (arrow). (C) Postoperative 3-dimensional reconstructive computerized tomography image showing incomplete cranioplasty (arrow indicates bone defect).

**Figure 3.**
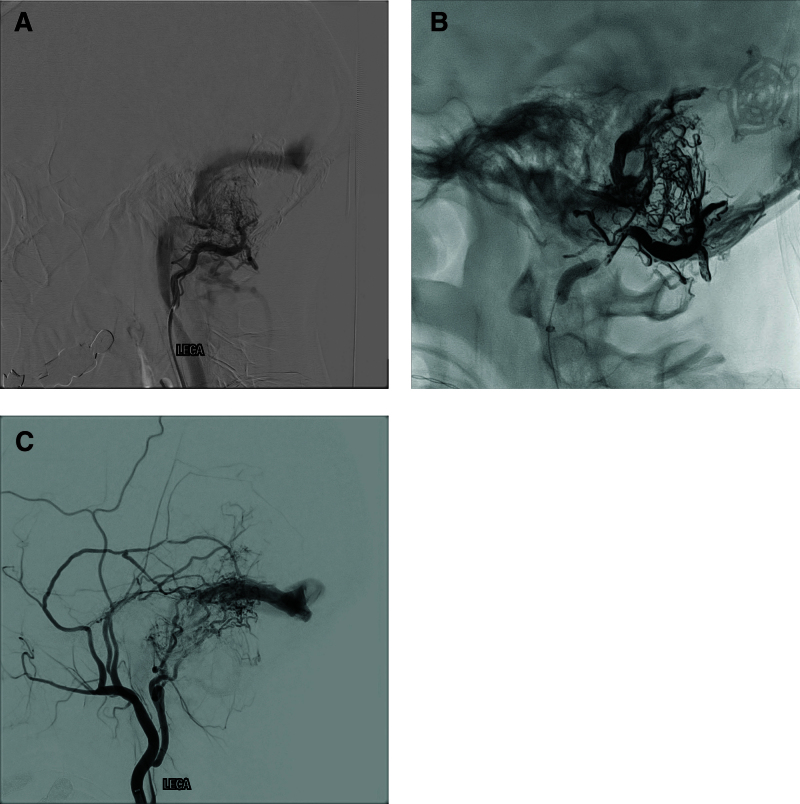
(A) Left external carotid artery angiography showing the dural arteriovenous fistula fed by the occipital artery. (B) Onyx® (Irvine, CA) embolization. (C) Digital subtraction angiography showing partial regression of the dural arteriovenous fistula.

A 51-year-old woman presented with headache, dizziness, and nausea that had persisted for several days. Initial brain magnetic resonance (MR) imaging showed a 40-mm-sized cerebellopontine angle mass with a mass effect (Fig. 1A, B). The patient underwent surgery via a retrosigmoid suboccipital approach. Small craniotomy and additional craniectomy were performed to expose the floor of the posterior fossa, sigmoid sinus, and transverse sinus. Sigmoid and transverse sinuses were partially exposed. After meningioma resection, the dura was closed in a watertight fashion without a muscle patch. The retrosigmoid bone flap measured 3 cm in size. After additional drilling to expose the venous sinus, the skull defect measured 4.5 cm. The bone flap was replaced and held in place using small titanium plates. However, complete cranioplasty was not performed using a bone substitute (Fig. 2A). Seven months postoperatively, the patient complained of operative site pulsation, bruit, and pulsating tinnitus. Follow-up MR imaging and MR angiography showed a dAVF without infarction or hemorrhage (Fig. 1C, D). Noncontrast time-of-flight images showed that numerous branches of the occipital artery were directly connected to the sigmoid sinus through the bony defect (Fig. 2B). Digital subtraction angiography showed a dAVF fed by the occipital artery. The dAVF drained into transverse and sigmoid sinuses (Fig. 3A). No sinus occlusion or sinus thrombus was observed. We performed Onyx® (Irvine, CA) embolization (Fig. 3B) and observed partial regression of the dAVF (Fig. 3C). The patient’s symptoms completely improved.

## 3. Discussion

We describe a case of iatrogenic dAVF after a retrosigmoid suboccipital craniotomy. dAVF is a heterogeneous disease. The mechanism underlying dAVF formation remains unknown. Secondary dAVFs resulting from trauma, sinus thrombosis, inflammation, and surgery have been reported.^[2–4]^ There are many reports of dAVF occurring after a ventriculostomy.^[5,6]^ However, only 4 cases of dAVF occurring after a rectosigmoid craniotomy or craniectomy have been reported.^[7–9]^

Schuette et al^[5]^ have reported a case of pail AVF from ventriculostomy. They showed pial AVF formation at the site of the previous ventriculostomy. The AVF resulted from cortical vessel injury during the procedure. Field et al^[6]^ have reported 2 cases of AVF fed by the middle meningeal artery after ventriculostomy. AVF formation occurred after a ventriculostomy in these studies, indicating that the cause of the AVF formation was a vascular injury during the procedure.^[5,6]^ Kim et al^[10]^ have reported dAVF formation resulting in the middle meningeal artery and middle meningeal vein injury during surgery.

In the present study, we carefully reviewed cases of dAVF that occurred after a retrosigmoid suboccipital craniotomy or craniectomy. Generally, a small craniotomy and an additional craniectomy are performed to expose the floor of the posterior fossa, sigmoid sinus, and transverse sinus. We performed a craniotomy with 2 burr holes. The first burr hole was away from venous sinuses to create a small bone flap. For the additional craniectomy, a part of the mastoid bone was drilled to expose the edge of the sigmoid sinus. A bony defect was created by craniotomy and additional craniectomy.

Many neurosurgeons prefer to perform an occipital craniectomy (approximately 2.5 cm in diameter) with a drill away from the occipital and part of the mastoid bone for cases with microvascular decompression (MVD) for hemifacial spasm (HFS) or trigeminal neuralgia. When craniectomy is performed for MVD, sigmoid and transverse sinuses are partially exposed. Reconstructive cranioplasty is generally performed for patients with craniectomy defects. Various cranioplasty methods have been reported for the reconstruction of a craniectomy defect in MVD.^[11]^ Numerous materials such as hydroxyapatite, titanium mesh, and polyetheretherketone are available for reconstructive cranioplasty.^[11]^

Small craniectomies for MVD are widely performed. Nabors et al^[8]^ have reported 2 cases of postoperative dAVFs, with suboccipital craniectomy performed for trigeminal neuralgia and HFS and Gelfoam placed over the bone defect. They did not perform complete cranioplasty. Several years later, dAVF at surgical site was diagnosed. Fed by the occipital artery, it drained into the sigmoid sinus.^[8]^ In both cases, cranioplasty was not performed to repair bone defect. In addition, the dAVF at surgery site was fed by the occipital artery. They suggested that arteriovenous shunt at the craniectomy site might have progressively developed to AVFs.^[8]^ Neovascularization can proceed to form a dAVF through a bone defect.

Sasaki et al^[9]^ have reported a case of dAVF after trigeminal neuroma surgery They used a transpetrosal and transtentorial approach for trigeminal neuroma. Sigmoid sinus and part of the transverse sinus were exposed.^[9]^ They did not perform complete cranioplasty. Postoperative computed tomography revealed a bone defect. Two years later, a dAVF at the surgical site was diagnosed. Fed by the occipital artery, it drained into the transverse sinus.^[9]^

Kim et al^[10]^ have reported a dAVF after a retrosigmoid craniectomy for HFS at surgical site. The patient did not undergo cranioplasty. Ten months later, a dAVF fed directly by the occipital artery was found.^[7]^ Computerized tomographic angiography and digital subtraction angiography showed a dAVF fed by the occipital artery through the bone defect.^[7]^

These 4 patients with dAVFs mentioned above underwent a small craniectomy. However, complete reconstructive cranioplasty was not performed. Directly fed by the occipital artery, these dAVFs drained into the sigmoid or transverse sinus through bone defects. Occipital arteries are directly connected to the transverse or sigmoid sinus. Finally, dAVFs are formed through bone defects.

We prefer to perform occipital craniotomy. However, removal of the mastoid bone is essential to expose transverse and sigmoid sinuses. Additional craniectomy allows a direct microscopic view of the petrous temporal bone. In our case, the retrosigmoid bone flap was 3 cm in size. After additional drilling to expose the sinus, the size of the skull defect was 4.5 cm. The bone flap was replaced and held in place by small titanium plates. However, complete cranioplasty was not performed using a bone substitute. Postoperative computed tomography revealed bone defect 1 cm above the sigmoid sinus (Fig. 2A, C). Eight months later, noncontrast time-of-flight imaging revealed that numerous branches of the occipital artery were directly connected to the sigmoid sinus through the bony defect (Fig. 2B). We could clearly observe that the exposed sinus was directly connected with occipital artery through a bone defect around the sinus. Craniectomy defects, partially exposed sinuses, and incomplete cranioplasty might be risk factors for iatrogenic dAVF after a retrosigmoid craniotomy or craniectomy. Complete cranioplasty is the substitution of the entire bone defect with either a bone flap or an artificial material. Complete bone repair may reduce the possibility of an iatrogenic dAVF after a craniotomy or craniectomy. Complete reconstructive cranioplasty is a surgical intervention that can prevent a direct connection between the venous sinus and the external carotid artery.

## 4. Conclusions

We describe a dAVF after a retrosigmoid suboccipital craniotomy through a bone defect. Craniectomy defects, partially exposed sinuses, and incomplete cranioplasty might be risk factors for an iatrogenic dAVF after a retrosigmoid suboccipital craniotomy or craniectomy. To prevent iatrogenic dAVF, complete reconstructive cranioplasty is essential to prevent direct connection between the venous sinus and the external carotid artery.

## Author contributions

Conceptualization: Jong-Myong Lee.

Formal analysis: Jung Soo Park.

Methodology: Jung Soo Park.

Writing – original draft: Jong-Myong Lee.

Writing – review&editing: Jung Soo Park, Jong-Myong Lee.
